# Redefining Trauma Triage for Elderly Adults: Development of Age-Specific Guidelines for Improved Patient Outcomes Based on a Machine-Learning Algorithm

**DOI:** 10.3390/medicina61050784

**Published:** 2025-04-23

**Authors:** Ji Yeon Lim, Yongho Jee, Seong Gyu Choi, Yoon Hee Choi, Sam S. Torbati, Carl T. Berdahl, Sun Hwa Lee

**Affiliations:** 1 Department of Emergency Medicine, Ewha Womans University Seoul Hospital, College of Medicine, Seoul 07804, Republic of Korea; jylim0923@ewha.ac.kr; 2 Advanced Biomedical Research Institute, Ewha Womans University Seoul Hospital, Seoul 07804, Republic of Korea; jyongho@ewha.ac.kr; 3 Institute for Health Promotion, Graduate School of Public Health, Yonsei University, Seoul 03722, Republic of Korea; csg526@naver.com; 4 Department of Emergency Medicine, Ewha Womans University Mokdong Hospital, College of Medicine, Seoul 03760, Republic of Korea; unii@ewha.ac.kr; 5 Department of Emergency Medicine, Cedars-Sinai Medical Center, Los Angeles, CA 90048, USA; sam.torbati@cshs.org (S.S.T.); carl.berdahl@csmc.edu (C.T.B.)

**Keywords:** age-specific triage guideline, elderly trauma patients, machine learning

## Abstract

*Background and Objectives*: Elderly trauma patients face unique physiological challenges that often lead to undertriage under the current guidelines. The present study aimed to develop machine-learning (ML)-based, age-specific triage guidelines to improve predictions for intensive care unit (ICU) admissions and in-hospital mortality. *Materials and Methods*: A total of 274,347 trauma cases transported via Emergency Medical System (EMS)-119 in Seoul (2020–2022) were analyzed. Physiological indicators (e.g., systolic blood pressure; saturation of partial pressure oxygen; and alert, verbal, pain, unresponsiveness scale) were incorporated. Bayesian optimization was used to fine-tuned models for sensitivity and specificity, emphasizing the F2 score to minimize undertriage. *Results*: Compared with the current guidelines, the alternative guidelines achieved superior sensitivity for ICU admissions (0.728 vs. 0.541) and in-hospital mortality (0.815 vs. 0.599). Subgroup analyses across injury severities, including traumatic brain and chest injuries, confirmed the enhanced performance of the alternative guidelines. *Conclusions*: ML-based, age-specific triage guidelines improve the sensitivity of triage decisions, reduce undertriage, and optimize elderly trauma care. Implementing these guidelines can significantly enhance patient outcomes and resource allocation in emergency settings.

## 1. Introduction

With the continuing aging of the global population, individuals aged 65 years and older are expected to constitute up to one-fifth of the global population by 2050, and this age group is estimated to account for nearly 39% of trauma admissions [1,2,3]. This demographic shift presents significant challenges, particularly for developed nations with higher life expectancies. This issue is particularly pressing in South Korea as the country is anticipated to become a superaged society by 2026, with over 20% of its population projected to be 65 years or older [4,5]. Undertriage and overtriage can be influenced by various contextual factors, including the availability of healthcare resources (such as the number of staffed high-cuity or ICU beds) and medicolegal considerations. As a result, field triage practices may differ across healthcare systems, depending on regional capabilities and policy environments. Despite these guidelines, some patients, particularly elderly patients, who appear stable in the field or emergency department (ED) still experience early mortality because of undertriage [6,7,8,9,10]. The contributing factors include altered physiological responses, low-impact injury mechanisms, comorbidities, frailty, and polypharmacy, all of which can also result in higher mortality, morbidity, and hospital costs [11,12,13,14]. According to the American Association for the Surgery of Trauma’s Geriatric Trauma Committee, elderly trauma patients are often undertriaged, and their outcomes are improved when they are admitted to higher-level trauma centers [15]. An undertriage rate below 5% and an overtriage rate of 25–50% are considered acceptable by the American College of Surgeons Committee on Trauma. However, a high overtriage rate can strain resources, while a high undertriage rate may lead to increased mortality due to failure in identifying severely injured patients [16].

Therefore, the present study aimed to develop age-specific trauma triage guidelines in Korea using machine-learning (ML) techniques. We seek to enhance the sensitivity of triage decisions for elderly patients by analyzing comprehensive trauma data. Current field triage guidelines define severe trauma by any of the following: systolic blood pressure below 90 mmHg, respiratory rate under 10 or over 29 breaths per minute, or a decreased level of consciousness (AVPU score of V or lower). However, these simplified thresholds may not sufficiently reflect the physiological variability seen in elderly patients [17]. We hypothesize that refining the triage criteria to better suit the needs of elderly trauma patients will improve their outcomes. Our research will suggest an age-specific triage guideline to ensure that emergency medical service personnel and emergency physicians can provide prompt medical interventions, leading to better prognosis for elderly trauma patients.

## 2. Materials and Methods

### 2.1. Current Triage Criteria for Severe Trauma [17]

#### Physiological Criteria

- Level of consciousness: AVPU scale with a score of “V” or lower, or a Glasgow Coma Scale score of 13 or lower.
- Systolic blood pressure below 90 mmHg.
- Respiratory rate: less than 10 breaths per minute or greater than 29 breaths per minute.

### 2.2. Study Population

#### 2.2.1. Study Setting and Data Source

The data for this study were sourced from the Seoul Golden Time Emergency Medical System (EMS) project, which includes records of patients transported to EDs across 25 medical institutions in Seoul via EMS-119 from 1 April 2020 to 31 December 2022. Seoul is the capital and largest city of South Korea, with a population of approximately 9 million in the city proper. It is one of the most populous and densely populated metropolitan areas worldwide. Most trauma patients in South Korea are transported by 119 emergency services, which are responsible for documenting transport records. These records contain basic patient information, vital signs, chief complaints, and initial physical examinations. The accuracy of these records is maintained through regular internal audits and reviews by supervisory physicians. The present study is significant because it integrates data from 119 transport services and receiving hospitals, combining previously separate datasets for the first time in South Korea. This multicenter study used data from 25 institutions across Seoul.

#### 2.2.2. Inclusion, Exclusion, and Preprocessing Criteria

Among the 274,537 patients who visited the ED from 2020 to 2022, we applied the following criteria for exclusion and grouping. First, we excluded patients with missing data for key variables (n = 53,819) including blood pressure, body temperature (BT), pulse rate (PR), respiratory rate (RR), and oxygen saturation. After this step, a total of 220,718 patients remained. Next, we removed participants with extreme outliers in the following key variables: systolic (SBP) or diastolic blood pressure (DBP) > 300 mmHg, BT > 50 °C or <20 °C, pulse rate (PR) > 300 beats per minute, RR > 50 breaths per minute, and oxygen saturation > 100% or <30%. This process led to the exclusion of an additional 1567 participants, resulting in a total of 219,151 participants who were eligible for further analysis.

#### 2.2.3. Dataset Construction and Group Stratification

**Figure 1:**
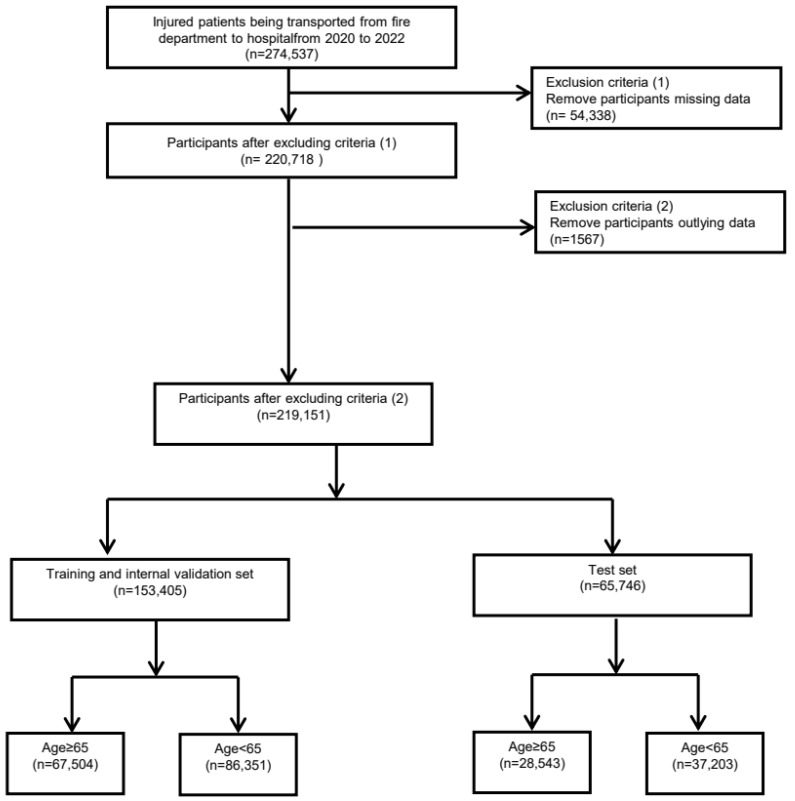
Flowchart of the study data set.

Subsequently, these participants were randomly divided into two sets: a training and internal validation set (n = 153,405) and a test set (n = 65,746). The patients in both sets were stratified by age (65 years and older or younger than 65 years). The training and internal validation set included 675,054 participants aged 65 years and older and 86,351 participants younger than 65 years. The test set included 28,543 participants aged 65 years and older and 37,203 participants younger than 65 years. The flowchart of study participants is shown in Figure 1.

#### 2.2.4. Triage Guideline Comparison and Final Stratification

We then compared the prediction performance among the current triage guidelines, alternative triage guidelines, and ML-based triage guidelines. The current triage guidelines were based on 10 in-hospital (IN) variables: age, sex, IN-AVPU (alert, verbal, pain, unresponsive) scale, IN_SBP, IN_DBP, PR, RR, BT, saturation of partial pressure oxygen (SpO_2_). We applied the ML-based guidelines and compared their performance on the same external validation set to compare the performance among the current, alternative, and ML-based guidelines. After selecting our final participants, we further stratified them by injury severity and specific injury types (e.g., traumatic brain injuries [TBIs], chest injuries, abdominal/pelvic injuries, and extremity injuries).

### 2.3. Measurement Variables

The key features selected for predicting intensive care unit (ICU) admission and in-hospital mortality included vital signs, such as SBP and DBP, PR, RR, BT, and oxygen saturation (SpO_2_). Moreover, the AVPU scale was used to assess patients’ initial level of consciousness. The injury severity score (ISS) was used to classify the severity of injuries. The Injury Severity Score (ISS) is an anatomical scoring system for evaluating multiple trauma severity. The body is divided into six regions, and each injury is scored using the Abbreviated Injury Scale (AIS), which rates injuries from 1 (minor) to 6 (unsurvivable) based on threat to life. ISS is calculated by summing the squares of the three highest AIS scores from the most severely injured regions [18]. These features were measured twice: once during the first fire department assessment and again during the in-hospital assessment. The outcomes were analyzed for both ICU admission and in-hospital mortality.

### 2.4. Statistical Analysis

#### 2.4.1. Strategies for Building Alternative Triage Guidelines

Bayesian optimization was used to identify optimal thresholds and combinations of features for triage guideline optimization [19]. This process involved determining specific thresholds (i.e., blood pressure below ‘threshold’ indicating severe trauma) and evaluating different feature combinations (i.e., both blood pressure and SpO_2_, or SpO_2_ alone) to enhance triage guideline performance. We derived a new feature by calculating the difference between first-stage fire service measurement variables and hospital measurement variables, representing changes in prognostic indicators during transport. This derived feature was used to refine the triage guidelines, enabling a more precise and systematic classification of patient conditions. The objective function for optimization was defined to maximize the F2 score, which prioritizes reducing false negatives (i.e., patients who are under triaged). The F2 score was chosen as the primary metric for objective function because it places greater weight on recall, which is critical in triage scenarios to avoid underestimating the severity of patients’ conditions. The triage guidelines were fine-tuned and validated on separate training (70% of the data) and testing datasets (30% of the data) for both age groups, and 5-fold cross validation was used to reduce bias and fine-tune the triage guidelines. The F2 score was calculated as follows:

F2 score = ((1 + β^2^) × (Precision × Recall))/(β^2^ × Precision + Recall)

#### 2.4.2. Strategies for Building ML-Based Triage Guidelines

To improve the sensitivity of severe trauma classification, we developed machine learning-based triage guidelines using logistic regression models with L1 and L2 regularization, specifically Lasso (L1 Norm) and Ridge (L2 Norm) regression. These models were used as baseline models for performance comparison due to their simplicity and interpretability.

In addition to the baseline models, we used a LightGBM model to develop a more sophisticated triage guideline.

This approach aimed to address the limitations of both the current and the alternative triage guidelines. While both triage guidelines are intuitive and easy to implement in the field, they rely on simple thresholds-based rules, which may over simplify the complexities of trauma and result in inaccurate classifications.

The model development process involved three steps: (1) selecting relevant variables, (2) fine-tuning hyper parameters to optimize model performance, and (3) evaluating model performance on a validation set before final optimization.

#### 2.4.3. Performance Evaluation

We used multiple performance metrics, including the F2 score, recall, specificity, and precision, to evaluate the triage guidelines. Accuracy was not used as the primary metric because of the highly imbalanced nature of the target variable (ICU admissions and in-hospital mortality). Instead, the F2 score was prioritized because it better captures the need to minimize false negatives in a triage context. The performance of these triage guidelines in predicting ICU admissions and in-hospital mortality was assessed across different patient groups, including those with high ISS (≥16) and specific injury types.

## 3. Results

### 3.1. Baseline Characteristics

The cohort was classified into two groups: those aged 65 years and older and those younger than 65 years (Table 1). The average age of the study population was 57.42 ± 22.04 years, with the elderly cohort (age ≥ 65 years) having an average age of 77.68 ± 7.92 years. The SBP at first assessment was higher in the elderly cohort (143.18 ± 29.39 mmHg) than in the younger cohort (131.68 ± 24.61 mmHg). Moreover, oxygen saturation (SpO_2_) was notably lower in the elderly cohort (96.10% ± 5.96%) than in the younger cohort (97.98% ± 3.20%).

### 3.2. Alternative Guidelines

We developed age-specific alternative guidelines for elderly trauma patients using an ML approach. The following are the key variables:

- SBP < 106 mmHg;
- SpO_2_ < 91%;
- RR < 8 or >22 breaths per minute;
- PR < 52 beats per minute;
- Decreased level of consciousness categorized as “V or below” (V, P, and U on the AVPU scale);
- Sudden change in consciousness level from alert (A) to unresponsive (U);
- Blood pressure variability ≥60 mmHg;
- Marked decrease in the PR of ≥44 beats per minute.

### 3.3. Prediction for ICU Admission

The alternative triage guidelines outperformed the current guidelines for predicting ICU admissions, particularly in elderly patients (age ≥ 65 years). As shown in Table 2, the alternative guidelines achieved a sensitivity and specificity of 0.728 and 0.693 for elderly patients, respectively, compared with the best performing current guidelines, which achieved a sensitivity and specificity of 0.541 and 0.84, respectively. Although the alternative guidelines demonstrated slightly lower precision (0.210), they provided a better balance by minimizing under triage while effectively managing over triage rates. Among younger patients (age < 65 years), the sensitivity of the alternative guidelines was 0.664, which is 16.7 percentage points higher than the best-performing current guideline (0.497). The specificity remained stable at 0.707, helping maintain a balance between over-triage and under-triage. While precision was slightly lower, the alternative guidelines demonstrated an overall improvement in patient selection compared to the current guidelines.

### 3.4. Prediction for In-Hospital Mortality

The alternative guidelines demonstrated higher sensitivity and balanced specificity for predicting in-hospital mortality among elderly patients. As shown in Table 2, the sensitivity improved to 0.815 compared with 0.599 under the current guidelines, whereas the specificity was maintained at 0.539. In younger patients (age < 65 years), the sensitivity and specificity improved to 0.828 and 0.593, respectively. These findings underscore the robustness of the alternative guidelines across age groups in addressing mortality risks (Table 3).

### 3.5. Subgroup Analysis by Injury Severity and Type

#### 3.5.1. High ISS (>16)

In elder patients with high ISS, the alternative guidelines showed superior performance for predicting ICU admission, achieving a sensitivity and specificity of 0.46 and 0.827 for elderly patients, respectively. These values represent a significant improvement over the current guidelines, which had a sensitivity of 0.376 (Supplementary Table S1). However, specificity was slightly lower at 0.827, compared to 0.94 in the best-performing current guideline. F1 Score and accuracy were also improved, indicating better identification of high-risk elderly trauma patients requiring ICU admission. For in-hospital mortality prediction: the alternative guidelines effectively improved sensitivity, ensuring that fewer critically ill elderly patients were under-triaged. Supplementary Table S2 presents the results for younger patients (<65 years) with ISS > 16. The sensitivity of the alternative guidelines for ICU admission prediction was 0.642, which is 17.9 percentage points higher than the best-performing current guideline (0.463). However, specificity decreased to 0.72, compared to 0.903 in the best-performing current guideline. Similar trends were observed in in-hospital mortality prediction, where the alternative guidelines achieved a sensitivity of 1.0, outperforming all current guidelines, but at the expense of specificity.

#### 3.5.2. TBIs

Among elderly patients with TBIs, the alternative guidelines achieved a sensitivity and specificity of 0.48 and 0.845, outperforming the current guidelines, which had a sensitivity and specificity of 0.409 and 0.936, respectively. Moreover, the sensitivity of the alternative guidelines in predicting in-hospital mortality within the TBI subgroup improved to 0.825, indicating enhanced predictive capacity for critical outcomes (Supplementary Table S3). Among elderly patients with TBIs, the alternative guidelines achieved a sensitivity and specificity of 0.48 and 0.845, outperforming the current guidelines, which had a sensitivity and specificity of 0.409 and 0.936, respectively. Moreover, the sensitivity of the alternative guidelines in predicting in-hospital mortality within the TBI subgroup improved to 0.825, indicating enhanced predictive capacity for critical outcomes (Supplementary Table S3). Supplementary Table S4 presents the results for younger patients (<65 years) with TBI. The sensitivity of the alternative guidelines for ICU admission prediction was 0.645, which is 12.9 percentage points higher than the best-performing current guideline (0.516). However, specificity decreased to 0.787, compared to 0.922 in the best-performing current guideline. In in-hospital mortality prediction, the sensitivity of the alternative guidelines reached 1.0, outperforming all current guidelines, but at the cost of slightly reduced specificity (0.638).

#### 3.5.3. Chest Injuries

As shown in Supplementary Table S5, the alternative triage guidelines demonstrated improved performance in predicting ICU admission among elderly patients (≥65 years) with chest injuries. The alternative triage guideline achieved a sensitivity of 0.278, which is higher than the best-performing current guideline (0.167), meaning that it better identifies high-risk patients requiring ICU admission. However, specificity dropped to 0.883, compared to 0.973 in the best-performing current guideline, leading to more false positives. From Supplementary Table S6, the test set results for ICU admission prediction in younger patients with chest injuries show that alternative guideline significantly improved sensitivity to 0.696. However, specificity decreased to 0.835, compared to 0.965 in the best-performing current guideline, meaning a higher false positive rate.

#### 3.5.4. Abdominal Pelvic Injury

From Supplementary Table S7, the test set results for ICU admission prediction in elderly patients with abdominal pelvic injuries show that the alternative triage guideline significantly improved sensitivity to 0.5, compared to 0.125 in all current guidelines, indicating better identification of critically severe patients. Supplementary Table S8, the test set results for ICU admission prediction in younger patients with abdominal pelvic injuries show the increased sensitivity in alternative guideline to 0.6.

#### 3.5.5. Extremity Injury Patients

From Supplementary Table S9, the test set results for ICU admission prediction in elderly patients with extremity injuries show higher sensitivity from alternative triage guideline (0.418) compared to the best-performing current guideline (0.327). For in-hospital mortality prediction in elderly patients, the alternative guideline achieved sensitivity of 0.471, compared to 0.324 in the best-performing current guideline, indicating better mortality detection. However, specificity dropped to 0.834, compared to 0.976 in the best-performing current guideline.

From Supplementary Table S10, the test set results for ICU admission prediction in younger patients with extremity injuries show the higher sensitivity among the alternative guideline (0.63) compared to 0.38 from the current guideline. Similarly, the specificity dropped to 0.733, compared to 0.936 in the best-performing current guideline, leading to more false positives.

## 4. Discussion

Many countries have established national standards for categorizing trauma patients for transfer to trauma centers, aiming to identify those at high risk for post trauma morbidity or mortality, maximize medical resource utilization, and minimize under triage rates through ongoing evaluation and quality improvement [20,21]. However, some patients who are deemed stable upon initial assessment or arrival at the ED may rapidly become unstable or die. Compared with younger patients, elderly patients have a less robust physiological response to physical stress, but their trauma severity and impairment are often underestimated [22,23]. The factors that contribute to undertriage include the lack of expected physiological responses (e.g., increased heart rate or decreased blood pressure), which could be because of physiological changes or chronic medications [11,12,13,14,15]. For example, beta-blockers that are used in hemorrhagic shock can diminish the standard compensatory heart rate increase. Similarly, baseline hypertensive patients may exhibit relative hypotension without meeting the hypotensive criteria. These conditions can lead to an underestimation of injury severity and delay the necessary aggressive treatment. Various reports suggest that up-triaging elderly patients is effective in reducing mortality and morbidity [8,24].

In South Korea, severe trauma cases must be transported to regional emergency medical centers based on specific physiological criteria, including Glasgow Coma Scale scores below 13, SBP below 90 mmHg, and abnormal RRs [17]. Research shows that the current triage guidelines present an ICU admission sensitivity and specificity of 0.394 and 0.893 for patients aged 65 years and older, with an in-hospital mortality sensitivity and specificity of 0.437 and 0.816, respectively. The alternative triage guidelines, which were optimized using ML algorithms, demonstrated superior performance in predicting ICU admissions and in-hospital mortality compared with the current guidelines, particularly for elderly patients whose physiological responses and injury presentations can significantly differ from those in the younger populations. This underscores the need for age-specific guidelines for elderly patients. Our study suggests that improving the identification of high-risk elderly patients in emergencies is possible through an age-specific alternative guideline based on objective physiological indicators using an ML approach. Considering that the current triage system is designed for all age groups, the age-specific guideline aims to avoid excessive complexity while remaining practical for field use.

Previous studies have highlighted that the standard adult field triage criteria have a low sensitivity for elderly trauma patients [23,24,25]. More moderate criteria, including a higher SBP threshold, have been shown to increase the sensitivity for trauma center needs and transport. For example, increasing the SBP cutoff from 90 mmHg to 110 mmHg reduced the undertriage rate from 6.9% to 3.2%. The predictors in the present study—age, SBP, consciousness level, and oxygen saturation—were selected based on clinical relevance and statistical validation to optimize accuracy while maintaining ease of use. Moreover, we proposed a model that is capable of predicting rapid changes in severity by incorporating a second set of measurements to assess changes in patient status. The alternative guidelines achieved an ICU admission prediction sensitivity and specificity of 0.728 and 0.693 and an in-hospital mortality prediction sensitivity and specificity of 0.815 and 0.539, respectively. For younger patients, the alternative guidelines’ sensitivity and specificity for predicting in-hospital mortality were 0.828 and 0.593, respectively, demonstrating the model’s effectiveness.

Our findings, supported by recent studies [26,27,28], suggest methods to better identify elderly patients with high-risk injuries. ML models offer a promising approach to refine triage guidelines, potentially leading to more effective decisions and improved patient outcomes. We can better identify those requiring urgent and specialized care by focusing on the unique characteristics of elderly patients. Our results demonstrate that up-triage guidelines should include age-specific criteria. Moreover, implementing ML-based models in clinical practice could enhance decision-making processes and reduce the risk of undertriage. In addition, ongoing training and education for emergency responders and medical personnel on the specific needs of elderly trauma patients are crucial.

Although this study is innovative as it integrated fire department and hospital data, it is limited to patients with matching records between the two sources, potentially introducing bias. Although comprehensive, the dataset is specific to Seoul and may not fully represent trauma care in other regions. However, integrating prehospital and in-hospital data represents a significant advancement in trauma care research, providing a more holistic view of patient management from injury to hospital admission. While the presence of missing data may have introduced potential bias, the substantial overall sample size is considered adequate to ensure the statistical reliability and robustness of the finding. Although we aimed to reflect ISS as an indicator of injury severity, the limited number of measurements prevented its inclusion in the alternative guidelines and showed no difference in sensitivity and specificity compared with the current guidelines. The age-specific alternative guideline, which was developed using an ML approach, may not fully capture age-related differences in real-world applications. Prospective evaluation and review in actual hospital settings are necessary for validating these guidelines. Furthermore, although ML models show promise, continuous validation and adjustment are needed to ensure effectiveness in diverse clinical scenarios.

## 5. Conclusions

This study highlights the critical need for age-specific triage guidelines to improve the management of elderly trauma patients. Our findings demonstrate that traditional triage criteria may be inadequate in identifying high-risk elderly patients, leading to potential undertriage and delayed treatment. By developing and evaluating alternative triage guidelines using machine-learning techniques, we have shown that these new guidelines offer improved sensitivity and specificity in predicting ICU admissions and posthospitalization mortality for elderly patients. The accuracy of triage decisions for elderly trauma patients can be improved by considering age-specific triage guidelines and leveraging ML approaches. Moreover, this shift has the potential to enhance patient outcomes, reduce mortality and morbidity, and optimize the use of medical resources. Further studies are warranted to prospectively evaluate the clinical impact of applying the triage guidelines used in this study to real-world EMS and ED settings.

## Figures and Tables

**Table 1:** Characteristics of injured patients transported by EMS stratified by age 65 years.

Demographics	All (n = 219,151)	Age ≥ 65 (n = 95,597)	Age < 65 (n = 123,554)
Age	57.42 ± 22.04	77.68 ± 7.92	41.74 ± 15.80
Sex (female)	109,909 (50.2%)	50,832 (53.2%)	59,077 (47.8%)
First Fire Department Assessment			
Systolic blood pressure	136.70 ± 27.40	143.18 ± 29.39	131.68 ± 24.61
Diastolic blood pressure	82.69 ± 17.38	83.13 ± 18.03	82.35 ± 16.86
Pulse rate	87.85 ± 19.35	86.65 ± 19.23	88.77 ± 19.39
Respiratory rate	18.08 ± 3.23	18.16 ± 3.40	18.02 ± 3.08
Body Temperature	36.89 ± 0.82	36.93 ± 0.91	36.85 ± 0.74
SpO_2_	97.16 ± 4.71	96.10 ± 5.96	97.98 ± 3.20
1st AVPU category			
A	201,644 (92.0%)	85,062 (89.0%)	116,582 (94.4%)
V	8465 (3.9%)	5032 (5.3%)	3433 (2.8%)
P	7408 (3.4%)	4576 (4.8%)	2832 (2.3%)
U	1634 (0.7%)	927 (1.0%)	707 (0.6%)
In-hospital Assessment			
Systolic blood pressure	138.18 ± 29.56	145.76 ± 31.49	132.30 ± 26.53
Diastolic blood pressure	79.68 ± 16.58	78.95 ± 16.76	80.24 ± 16.41
Pulse rate	86.44 ± 19.84	85.30 ± 19.96	87.32 ± 19.70
Respiratory rate	19.37 ± 2.93	19.61 ± 3.25	19.18 ± 2.64
Body Temperature	36.77 ± 0.78	36.77 ± 0.86	36.77 ± 0.72
SpO_2_	97.25 ± 3.59	96.59 ± 4.24	97.83 ± 2.77
2nd AVPU category			
A	200,625 (91.5%)	84,186 (88.1%)	116,439 (94.2%)
V	10,391 (4.7%)	6323 (6.6%)	4068 (3.3%)
P	7382 (3.4%)	4625 (4.8%)	2757 (2.2%)
U	753 (0.3%)	463 (0.5%)	290 (0.2%)
Korean Triage and Acuity Scale			
Level 1, Resuscitation	2084 (1.0%)	1356 (1.4%)	728 (0.6%)
Level 2, Emergent	29,030 (13.2%)	16,055 (16.8%)	12,975 (10.5%)
Level 3, Urgent	114,763 (52.4%)	52,837 (55.3%)	61,926 (50.1%)
Level 4, Less Urgent	69,119 (31.5%)	23,866 (25.0%)	45,253 (36.6%)
Level 5, Non Urgent	4152 (1.9%)	1481 (1.5%)	2671 (2.2%)
Injury Severity Score			
ISS > 16	7067 (65.4%)	4157 (73.9%)	2910 (56.1%)
ISS < 16	3746 (34.6%)	1470 (26.1%)	2276 (43.9%)
Type of Discharge			
Home Discharge	149,087 (68.1%)	52,813 (55.3%)	96,274 (78.0%)
Transferred	7133 (3.3%)	4276 (4.5%)	2857 (2.3%)
General Ward Admission	45,191 (20.6%)	27,882 (29.2%)	17,309 (14.0%)
ICU Admission	15,417 (7.0%)	9565 (10.0%)	5852 (4.7%)
Other Admission	72 (0.0%)	10 (0.0%)	62 (0.1%)
Dead on Arrival	17 (0.0%)	15 (0.0%)	2 (0.0%)
Other Death	616 (0.3%)	505 (0.5%)	111 (0.1%)
Other	996 (0.5%)	342 (0.4%)	654 (0.5%)
Unknown	410 (0.2%)	115 (0.1%)	295 (0.2%)
Post-Hospitalization Outcomes			
Discharged in Stable Condition	44,470 (77.3%)	25,910 (73.2%)	18,560 (84.1%)
Discharged Against Medical Advice	1641 (2.9%)	906 (2.6%)	735 (3.3%)
Transferred	6702 (11.7%)	4908 (13.9%)	1794 (8.1%)
Death	4529 (7.9%)	3597 (10.2%)	932 (4.2%)
Absconded	45 (0.1%)	11 (0.0%)	34 (0.2%)
Discharged with Poor Prognosis	17 (0.0%)	13 (0.0%)	4 (0.0%)
Other	96 (0.2%)	74 (0.2%)	22 (0.1%)

ICU: intensive care unit.

**Table 2:** Comparative accuracy of alternative triage guidelines versus current guidelines in predicting ICU admission and post-hospitalization mortality among patients aged 65 and older.

**Target**	**ICU Admission**											
	**Training Set (n = 66,555)**						**Test Set (n = 28,407)**					
**All, Age ≥ 65**	**Current** **Triage** **Guideline 1 ^1^**	**Current** **Triage** **Guideline 2 ^2^**	**Current** **Triage** **Guidelines 3 ^3^**	**Alternative** **Triage Guideline**	**Light** **GBM**	**Lasso**	**Current** **Triage Guideline 1**	**Current Triage Guideline 2**	**Current** **Triage** **Guideline 3**	**Alternative** **Triage Guideline**	**Light** **GBM**	**Lasso**
Sensitivity	0.394	0.470	0.544	0.734	0.798	0.71	0.397	0.461	0.541	0.728	0.787	0.696
Specificity	0.893	0.887	0.838	0.693	0.697	0.701	0.892	0.887	0.84	0.693	0.699	0.704
Precision	0.291	0.317	0.274	0.211	0.228	0.210	0.292	0.314	0.275	0.210	0.227	0.209
F1 Score	0.335	0.379	0.364	0.328	0.354	0.324	0.336	0.373	0.365	0.326	0.353	0.322
F2 score	0.368	0.429	0.454	0.491	0.531	0.481	0.370	0.422	0.453	0.487	0.527	0.475
Accuracy	0.843	0.845	0.809	0.697	0.707	0.702	0.842	0.844	0.81	0.696	0.708	0.704
**Target**	**Post-Hospitalization Mortality**											
	**Training Set (n = 24,815)**						**Test Set (n = 10,604)**					
**All, Age ≥ 65**	**Current** **Triage** **Guideline 1 ^1^**	**Current** **Triage** **Guideline 2 ^2^**	**Current** **Triage** **Guidelines 3 ^3^**	**Alternative** **Triage Guideline**	**Light** **GBM**	**Lasso**	**Current** **Triage Guideline 1 ^1^**	**Current Triage Guideline 2**	**Current** **triage** **Guideline 3**	**Alternative** **Triage Guideline**	**Light** **GBM**	**Lasso**
Sensitivity	0.437	0.513	0.600	0.812	0.803	0.738	0.471	0.503	0.599	0.815	0.812	0.74
Specificity	0.816	0.787	0.727	0.537	0.699	0.695	0.816	0.789	0.729	0.539	0.702	0.695
Precision	0.212	0.214	0.200	0.166	0.232	0.215	0.223	0.211	0.199	0.165	0.234	0.214
F1 Score	0.286	0.302	0.300	0.276	0.3	0.333	0.303	0.297	0.298	0.275	0.363	0.332
F2 score	0.361	0.401	0.428	0.457	0.538	0.497	0.385	0.394	0.427	0.456	0.543	0.496
Accuracy	0.777	0.759	0.714	0.565	0.709	0.699	0.781	0.760	0.716	0.567	0.713	0.700

^1^ using First Fire Department Assessment variables. ^2^ using In-hospital Assessment ^3^ criteria are meeting either ^1^ or ^2^ exceeds the threshold.

**Table 3:** Comparative accuracy of alternative triage guidelines versus current guidelines in predicting ICU admission and post-hospitalization among patients under 65 years old.

**Target**	**ICU Admission**											
	**Training Set (n = 86,120)**						**Test Set (n = 37,026)**					
**All, Age < 65**	**Current** **Triage** **Guideline 1 ^1^**	**Current** **Triage** **Guideline 2 ^2^**	**Current** **Triage** **Guidelines 3 ^3^**	**Alternative** **Triage Guidelines**	**Light** **GBM**	**Lasso**	**Current Triage** **Guideline 1**	**Current Triage** **Guideline 2**	**Current Triage** **Guidelines 3**	**Alternative Triage Guidelines**	**Light** **GBM**	**Lasso**
Sensitivity	0.353	0.414	0.486	0.657	0.881	0.766	0.356	0.428	0.497	0.664	0.865	0.746
Specificity	0.932	0.934	0.897	0.707	0.699	0.701	0.932	0.933	0.896	0.707	0.7	0.703
Precision	0.206	0.238	0.191	0.101	0.127	0.113	0.208	0.241	0.193	0.101	0.126	0.111
F1 Score	0.26	0.303	0.274	0.174	0.223	0.197	0.263	0.309	0.278	0.176	0.22	0.194
F2 score	0.309	0.361	0.371	0.312	0.403	0.356	0.312	0.371	0.378	0.315	0.397	0.348
Accuracy	0.904	0.909	0.878	0.704	0.707	0.704	0.905	0.909	0.877	0.705	0.708	0.705
**Target**	**Post-Hospitalization Mortality**											
	**Training Set (n = 15,435)**						**Test Set (n = 6646)**					
**All, Age < 65**	**Current** **Triage** **Guideline 1 ^1^**	**Current** **Triage** **Guideline 2 ^2^**	**Current** **Triage** **Guidelines 3 ^3^**	**Alternative** **Triage Guidelines**	**Light** **GBM**	**Lasso**	**Current Triage** **Guideline 1 ^1^**	**Current Triage** **Guideline 2 ^2^**	**Current Triage** **Guidelines 3**	**Alternative Triage Guidelines**	**Light** **GBM**	**Lasso**
Sensitivity	0.414	0.523	0.587	0.802	0.939	0.801	0.448	0.597	0.655	0.828	0.941	0.821
Specificity	0.855	0.844	0.791	0.595	0.695	0.697	0.861	0.846	0.796	0.593	0.706	0.71
Precision	0.11	0.127	0.109	0.079	0.118	0.103	0.128	0.151	0.128	0.085	0.128	0.114
F1 Score	0.174	0.205	0.312	0.144	0.21	0.183	0.199	0.24	0.214	0.154	0.225	0.201
F2 score	0.267	0.322	0.183	0.284	0.393	0.34	0.299	0.375	0.359	0.301	0.414	0.367
Accuracy	0.836	0.831	0.782	0.603	0.705	0.702	0.843	0.836	0.79	0.603	0.716	0.715

^1^ using First Fire Department Assessment variables. ^2^ using In-hospital Assessment ^3^ criteria are meeting either ^1^ or ^2^ exceeds the threshold.

## Data Availability

The data presented in this study are available on request from the corresponding author.

## Supplementary Materials

The following supporting information can be downloaded at: https://www.mdpi.com/article/10.3390/medicina61050784/s1, Table S1: Comparative Accuracy of Alternative Triage Guidelines Versus Current Guidelines in Predicting ICU Admission and Post-hospitalization mortality for Patients Aged 65 and older with High Injury Severity Score (ISS > 16); Table S2: Comparative Accuracy of Alternative Triage Guidelines Versus Current Guidelines in Predicting ICU Admission and Post-hospitalization mortality for patients aged under 65-year-old with high Injury Severity Score (ISS > 16); Table S3: Comparative Accuracy of Alternative Triage Guidelines Versus Current Guidelines in Predicting ICU Admission for Patients aged 65 and Older with Traumatic Brain Injury (TBI); Table S4: Comparative Accuracy of Alternative Triage Guidelines Versus Current Guidelines in Predicting ICU Admission for patients aged under 65-year-old with Traumatic Brain Injury (TBI); Table S5: Comparative Accuracy of Alternative Triage Guidelines Versus Current Guidelines in Predicting ICU Admission for Patients Aged 65 and Older with Chest injury; Table S6: Comparative Accuracy of Alternative Triage Guidelines Versus Current Guidelines in Predicting ICU Admission for patients aged under 65-year-old Chest injury; Table S7: Comparative Accuracy of Alternative Triage Guidelines Versus Current Guidelines in Predicting ICU Admission for Patients Aged 65 and Older with Abdominal Pelvic injury; Table S8: Comparative Accuracy of Alternative Triage Guidelines Versus Current Guidelines in Predicting Post-hospitalization mortality for Patients Aged under 65 years old with Abdominal Pelvic injury; Table S9: Comparative Accuracy of Alternative Triage Guidelines Versus Current Guidelines in Predicting ICU Admission for Patients Aged 65 and older with extremity; Table S10: Comparative Accuracy of Alternative Triage Guidelines Versus Current Guidelines in Predicting Post-hospitalization mortality for Patients Aged under 65 years old with extremity.

## Abbreviations

AVPU	alert, verbal, pain, unresponsive
BT	body temperature
ED	emergency department
EMS	emergency medical system
DBP	diastolic blood pressure
FD	fire department
ICU	intensive care unit
IN	in-hospital
ISS	injury severity score
ML	machine-learning
SBP	systolic blood pressure
SpO_2_	saturation of partial pressure oxygen
TBIs	traumatic brain injuries

## References

[1] Adams S.D., Holcomb J.B. (2015). Geriatric trauma. Curr. Opin. Crit. Care.

[2] Department of Economic and Social Affairs Population Division (2002). World Population Ageing: 1950–2050.

[3] Vespa J. The US Joins Other Countries with Large Aging Populations. United States Census Bureau. https://www.census.gov/library/stories/2018/03/graying-america.html.

[4] Jung H.-W., Lim J.-Y. (2018). Geriatric medicine, an underrecognized solution of precision medicine for older adults in Korea. Ann. Geriatr. Med. Res..

[5] Lee Y., Kim S., Hwang N., Im J., Joo B., Namgung E., Lee S., Jung K., Kang E., Kim G. (2020). Examining the Status of the Older Adults in 2020.

[6] Brown E., Tohira H., Bailey P., Fatovich D., Pereira G., Finn J. (2019). Older age is associated with a reduced likelihood of ambulance transport to a trauma centre after major trauma in Perth. Emerg. Med. Australas..

[7] Cox S., Morrison C., Cameron P., Smith K. (2014). Advancing age and trauma: Triage destination compliance and mortality in Victoria, Australia. Injury.

[8] Garwe T., Stewart K., Stoner J., Newgard C.D., Scott M., Zhang Y., Cathey T., Sacra J., Albrecht R.M. (2017). Out-of-hospital and inter-hospital under-triage to designated tertiary trauma centers among injured older adults: A 10-year statewide geospatial-adjusted analysis. Prehospital Emerg. Care.

[9] Meyers M.H., Wei T.L., Cyr J.M., Hunold T.M., Shofer F.S., Cowden C.S., Moss C.F., Jensen C.E., Platts-Mills T.F., Brice J.H. (2019). The triage of older adults with physiologic markers of serious injury using a state-wide prehospital plan. Prehospital Disaster Med..

[10] Horst M.A., Jammula S., Gross B.W., Cook A.D., Bradburn E.H., Altenburg J., Von Nieda D., Morgan M., Rogers F.B. (2018). Undertriage in trauma: Does an organized trauma network capture the major trauma victim? A statewide analysis. J. Trauma Acute Care Surg..

[11] Aalami O.O., Fang T.D., Song H.M., Nacamuli R.P. (2003). Physiological features of aging persons. Arch. Surg..

[12] Faller J.W., Pereira D.d.N., de Souza S., Nampo F.K., Orlandi F.d.S., Matumoto S. (2019). Instruments for the detection of frailty syndrome in older adults: A systematic review. PLoS ONE.

[13] Haas B., Wunsch H. (2016). How does prior health status (age, comorbidities and frailty) determine critical illness and outcome?. Curr. Opin. Crit. Care.

[14] Brown C.V., Rix K., Klein A.L., Ford B., Teixeira P.G., Aydelotte J., Coopwood B., Ali S. (2016). A comprehensive investigation of comorbidities, mechanisms, injury patterns, and outcomes in geriatric blunt trauma patients. Am. Surg..

[15] Egodage T., Ho V.P., Bongiovanni T., Knight-Davis J., Adams S.D., Digiacomo J., Swezey E., Posluszny J., Ahmed N., Prabhakaran K. (2024). Geriatric trauma triage: Optimizing systems for older adults—A publication of the American Association for the Surgery of Trauma Geriatric Trauma Committee. Trauma Surg. Acute Care Open.

[16] van Laarhoven J., Lansink K., van Heijl M., Lichtveld R., Leenen L. (2014). Accuracy of the field triage protocol in selecting severely injured patients after high energy trauma. Injury.

[17] 119 Emergency Medical Technician Field First Aid Standard Guidelines (2023 Revised Edition). https://www.nfa.go.kr/nfa/publicrelations/legalinformation/archives/?boardId=bbs_0000000000000018&mode=view&cntId=50&category=&pageIdx=&searchCondition=&searchKeyword=.

[18] Baker S.P., O’Neill B., Haddon W., Long W.B. (1974). The Injury Severity Score: A method for describing patients with multiple injuries and evaluating emergency care. J. Trauma.

[19] Akiba T., Sano S., Yanase T., Ohta T., Koyama M. Optuna: A Next-Generation Hyperparameter Optimization Framework. Proceedings of the 25th ACM SIGKDD International Conference on Knowledge Discovery & Data Mining.

[20] Maughan B.C.M., Lin A., Caughey A.B., Bulger E.M.M., McConnell K.J., Malveau S., Griffiths D.B., Newgard C.D. (2022). Field trauma triage among older adults: A cost-effectiveness analysis. J. Am. Coll. Surg..

[21] Morris R.S., Karam B.S., Murphy P.B., Jenkins P., Milia D.J., Hemmila M.R., Haines K.L., Puzio T.J., de Moya M.A., Tignanelli C.J. (2021). Field-triage, hospital-triage and triage-assessment: A literature review of the current phases of adult trauma triage. J. Trauma Acute Care Surg..

[22] (2012). Trauma ACoSCo. ACS TQIP Geriatric Trauma Management Guidelines. https://www.facs.org/media/rddahzbb/geriatric_guidelines.pdf.

[23] Alshibani A., Alharbi M., Conroy S. (2021). Under-triage of older trauma patients in prehospital care: A systematic review. Eur. Geriatr. Med..

[24] Weber C., Millen J.C., Liu H., Clark J., Ferber L., Richards W., Ang D. (2022). Undertriage of geriatric trauma patients in Florida. J. Surg. Res..

[25] Alshibani A., Singler B., Conroy S. (2021). Towards improving prehospital triage for older trauma patients. Z. Gerontol. Geriatr..

[26] Huang C.Y., Wu S.C., Lin T.S., Kuo P.J., Yang J.C., Hsu S.Y., Hsieh C.H. (2024). Efficacy of the Geriatric Trauma Outcome Score (GTOS) in Predicting Mortality in Trauma Patients: A Retrospective Cross-Sectional Study. Diagnostics.

[27] Zhuang Y., Feng Q., Tang H., Wang Y., Li Z., Bai X. (2022). Predictive Value of the Geriatric Trauma Outcome Score in Older Patients After Trauma: A Retrospective Cohort Study. Int. J. Gen. Med..

[28] Cubitt M., Key R. (2023). Geriatric Trauma Triage—The Scope of the Problem. J. Geriatr. Emerg. Med..

