# Supplementary material for: Redefining Trauma Triage for Elderly Adults: Development of Age-Specific Guidelines for Improved Patient Outcomes Based on a Machine-Learning Algorithm
Source: Medicina (Kaunas). 2025 Apr 23;61(5):784. doi: 10.3390/medicina61050784 (PMC12113527; doi:10.3390/medicina61050784)
Supplement: Supplementary file 1 [file medicina-61-00784-s001.zip › medicina-3549335-supplementary.pdf]

Supplementary Table S1. Comparative Accuracy of Alternative Triage Guidelines Versus Current Guidelines in Predicting ICU Admission and Post-hospitalization mortality for Patients Aged 65 and older with High Injury Severity Score (ISS > 16)

Target	ICU admission											
	Training set (n=2,899)						Test set (n=1,241)					
ISS≥16, age≥65	Current triage Guideline1[1]	Current triage Guideline 2[2]	Current triage Guidelines 3[3]	Alternative triage guideline	Light GBM	Lasso	Current triage guideline1	Current triage guideline2	Current triage guideline3	Alternative triage guideline	Light GBM	Lasso
Sensitivity	0.328	0.352	0.42	0.537	0.619	0.543	0.266	0.338	0.376	0.46	0.54	0.515
Specificity	0.967	0.954	0.937	0.835	0.841	0.844	0.965	0.96	0.94	0.827	0.863	0.849
Precision	0.667	0.61	0.574	0.397	0.44	0.413	0.643	0.667	0.597	0.385	0.481	0.445
F1 Score	0.44	0.447	0.485	0.456	0.514	0.469	0.376	0.448	0.461	0.419	0.509	0.477
F2 score	0.365	0.385	0.444	0.502	0.572	0.511	0.301	0.375	0.406	0.443	0.527	0.499
Accuracy	0.859	0.853	0.85	0.785	0.803	0.793	0.832	0.841	0.832	0.757	0.801	0.785
	Post-hospitalization mortality											
	Training set (n=2,409)						Test set (n=1,039)					
ISS≥16, age≥65	Current triage Guideline1[1]	Current triage Guideline 2[2]	Current triage Guidelines 3[3]	Alternative triage guideline	Light GBM	Lasso	Current triage guideline1	Current triage guideline2	Current triage guideline3	Alternative triage guideline	Light GBM	Lasso
Sensitivity	0.39	0.468	0.511	0.61	0.582	0.482	0.412	0.451	0.49	0.529	0.588	0.431
Specificity	0.934	0.93	0.904	0.802	0.896	0.891	0.94	0.928	0.906	0.793	0.885	0.876
Precision	0.268	0.295	0.248	0.16	0.258	0.216	0.262	0.245	0.212	0.116	0.208	0.152
F1 Score	0.318	0.362	0.334	0.254	0.357	0.298	0.321	0.317	0.296	0.191	0.308	0.224
F2 score	0.358	0.419	0.422	0.391	0.465	0.387	0.37	0.386	0.388	0.31	0.431	0.315
Accuracy	0.902	0.903	0.881	0.79	0.878	0.867	0.914	0.905	0.885	0.78	0.87	0.854

1) using First Fire Department Assessment variables. 2) using In-hospital Assessment 3) criteria arte meeting either 1) or 2) exceeds the threshold

Supplementary Table S2. Comparative Accuracy of Alternative Triage Guidelines Versus Current Guidelines in Predicting ICU Admission and Post-hospitalization mortality for patients aged under 65-year-old with high Injury Severity Score (ISS > 16)

Target	ICU admission											
	Training set (n= 2,055)						Test set (n= 845)					
ISS≥16, age<65	Current triage Guideline1[1]	Current triage Guideline 2[2]	Current triage Guidelines 3[3]	Alternative triage guideline	Light GBM	Lasso	Current triage guideline1	Current triage guideline2	Current triage guideline3	Alternative triage guideline	Light GBM	Lasso
Sensitivity	0.361	0.406	0.472	0.613	0.81	0.709	0.354	0.382	0.463	0.642	0.801	0.675
Specificity	0.92	0.917	0.883	0.734	0.579	0.563	0.932	0.925	0.903	0.72	0.613	0.589
Precision	0.635	0.654	0.609	0.472	0.427	0.386	0.68	0.676	0.663	0.485	0.459	0.403
F1 Score	0.46	0.501	0.532	0.533	0.56	0.5	0.465	0.488	0.545	0.552	0.584	0.505
F2 score	0.395	0.439	0.494	0.579	0.687	0.607	0.391	0.419	0.493	0.603	0.697	0.595
Accuracy	0.764	0.774	0.768	0.7	0.644	0.604	0.763	0.767	0.775	0.697	0.667	0.614
	Post-hospitalization mortality											
	Training set (n= 1,687)						Test set (n= 714)					
ISS≥16, age<65	Current triage Guideline1[1]	Current triage Guideline2[2]	Current triage Guidelines 3[3]	Alternative triage guideline	Light GBM	Lasso	Current triage guideline1	Current triage guideline2	Current triage guideline3	Alternative triage guideline	Light GBM	Lasso
Sensitivity	0.676	0.853	0.882	0.941	0.971	0.882	1	1	1	1	1	0.933
Specificity	0.864	0.858	0.817	0.668	0.753	0.717	0.867	0.866	0.821	0.641	0.775	0.754
Precision	0.093	0.11	0.09	0.055	0.075	0.06	0.139	0.138	0.107	0.056	0.087	0.075
F1 Score	0.163	0.195	0.164	0.104	0.139	0.113	0.244	0.242	0.194	0.107	0.16	0.139
F2 score	0.299	0.363	0.321	0.223	0.285	0.237	0.446	0.444	0.375	0.23	0.323	0.285
Accuracy	0.86	0.858	0.819	0.674	0.757	0.72	0.87	0.868	0.825	0.648	0.78	0.758

1) using First Fire Department Assessment variables. 2) using In-hospital Assessment 3) criteria meeting either 1) or 2) exceeds the threshold

Supplementary table S3. Comparative Accuracy of Alternative Triage Guidelines Versus Current Guidelines in Predicting ICU Admission for Patients aged 65 and Older with Traumatic Brain Injury (TBI)

Target	ICU admission											
	Training set (n=5,998)						Test set (n=2,576)					
TBI, age≥65	Current triage Guideline1[1]	Current triage Guideline 2[2]	Current triage Guidelines 3[3]	Alternative triage guideline	Light GBM	Lasso	Current triage guideline1	Current triage guideline2	Current triage guideline3	Alternative triage guideline	Light GBM	Lasso
Sensitivity	0.315	0.368	0.432	0.518	0.623	0.535	0.262	0.387	0.409	0.48	0.56	0.524
Specificity	0.967	0.942	0.924	0.843	0.821	0.787	0.976	0.946	0.936	0.845	0.83	0.796
Precision	0.417	0.326	0.302	0.2	0.209	0.16	0.484	0.385	0.357	0.213	0.224	0.184
F1 Score	0.359	0.345	0.355	0.288	0.313	0.246	0.34	0.386	0.381	0.295	0.32	0.272
F2 score	0.331	0.359	0.397	0.393	0.446	0.364	0.289	0.386	0.397	0.384	0.431	0.382
Accuracy	0.921	0.902	0.89	0.82	0.808	0.769	0.918	0.901	0.893	0.816	0.809	0.774
	ICU admission											
	Training set (n=1,087)						Test set (n=495)					
TBI , age≥65	Current triage Guideline1[1]	Current triage Guideline2[2]	Current triage Guidelines3[3]	Alternative triage guideline	Light GBM	Lasso	Current triage guideline1	Current triage guideline2	Current triage guideline3	Alternative triage guideline	Light GBM	Lasso
Sensitivity	0.533	0.609	0.685	0.728	0.728	0.565	0.6	0.75	0.775	0.825	0.8	0.625
Specificity	0.885	0.844	0.81	0.712	0.835	0.842	0.899	0.851	0.826	0.716	0.831	0.84
Precision	0.301	0.265	0.25	0.189	0.29	0.249	0.343	0.306	0.282	0.204	0.294	0.255
F1 Score	0.384	0.37	0.366	0.3	0.415	0.346	0.436	0.435	0.413	0.327	0.43	0.362
F2 score	0.461	0.484	0.508	0.464	0.559	0.451	0.522	0.581	0.574	0.512	0.595	0.484
Accuracy	0.856	0.824	0.799	0.713	0.826	0.819	0.875	0.842	0.822	0.725	0.828	0.822

1) using First Fire Department Assessment variables. 2) using In-hospital Assessment 3) criteria meeting either 1) or 2) exceeds the threshold

Supplementary table S4. Comparative Accuracy of Alternative Triage Guidelines Versus Current Guidelines in Predicting ICU Admission for patients aged under 65-year-old with Traumatic Brain Injury (TBI)

Target	ICU admission											
	Training set (n= 6,658)						Test set (n=2,903)					
TBI , age<65	Current triage Guideline1[1]	Current triage Guideline 2[2]	Current triage Guidelines 3[3]	Alternative triage guideline	Light GBM	Lasso	Current triage guideline1	Current triage guideline2	Current triage guideline3	Alternative triage guideline	Light GBM	Lasso
Sensitivity	0.421	0.466	0.534	0.638	0.826	0.761	0.439	0.426	0.516	0.645	0.813	0.748
Specificity	0.961	0.939	0.921	0.785	0.653	0.608	0.96	0.943	0.922	0.787	0.653	0.618
Precision	0.38	0.303	0.277	0.143	0.118	0.099	0.384	0.296	0.272	0.146	0.117	0.099
F1 Score	0.399	0.367	0.365	0.234	0.207	0.175	0.41	0.349	0.356	0.238	0.204	0.175
F2 score	0.412	0.421	0.45	0.377	0.376	0.325	0.427	0.391	0.438	0.383	0.371	0.325
Accuracy	0.932	0.914	0.901	0.777	0.662	0.616	0.932	0.915	0.9	0.78	0.662	0.625
	Post-hospitalization mortality											
	Training set (n=853)						Test set (n=377)					
TBI , age<65	Current triage Guideline1[1]	Current triage Guideline2[2]	Current triage Guidelines3[3]	Alternative triage guideline	Light GBM	Lasso	Current triage guideline1	Current triage guideline2	Current triage guideline3	Alternative triage guideline	Light GBM	Lasso
Sensitivity	0.771	0.8	0.829	0.914	0.943	0.914	0.867	0.933	0.933	1	1	0.933
Specificity	0.824	0.803	0.758	0.642	0.72	0.66	0.818	0.82	0.765	0.638	0.74	0.685
Precision	0.158	0.148	0.128	0.098	0.126	0.103	0.165	0.177	0.141	0.103	0.138	0.109
F1 Score	0.262	0.25	0.221	0.178	0.222	0.186	0.277	0.298	0.246	0.186	0.242	0.196
F2 score	0.434	0.426	0.395	0.344	0.41	0.356	0.468	0.504	0.44	0.364	0.444	0.372
Accuracy	0.822	0.803	0.761	0.653	0.729	0.671	0.82	0.825	0.772	0.653	0.751	0.695

1) using First Fire Department Assessment variables. 2) using In-hospital Assessment 3) criteria meeting either 1) or 2) exceeds the threshold

Supplementary table S5. Comparative Accuracy of Alternative Triage Guidelines Versus Current Guidelines in Predicting ICU Admission for Patients Aged 65 and Older with Chest injury

Target	ICU admission											
	Training set (n= 547)						Test set (n= 240)					
Chest , age≥65	Current triage Guideline1[1]	Current triage Guideline 2[2]	Current triage Guidelines 3[3]	Alternative triage guideline	Light GBM	Lasso	Current triage guideline1	Current triage guideline2	Current triage guideline3	Alternative triage guideline	Light GBM	Lasso
Sensitivity	0.189	0.297	0.378	0.622	0.676	0.649	0.056	0.167	0.167	0.278	0.389	0.444
Specificity	0.982	0.975	0.965	0.843	0.841	0.814	1	0.973	0.973	0.883	0.869	0.874
Precision	0.438	0.458	0.438	0.223	0.236	0.202	1	0.333	0.333	0.161	0.194	0.222
F1 Score	0.264	0.361	0.406	0.329	0.35	0.308	0.105	0.222	0.222	0.204	0.259	0.296
F2 score	0.213	0.32	0.389	0.458	0.492	0.449	0.068	0.185	0.185	0.243	0.324	0.37
Accuracy	0.929	0.929	0.925	0.828	0.83	0.803	0.929	0.912	0.912	0.838	0.833	0.842
	Post-hospitalization mortality											
	Training set (n=149)						Test set (n=70)					
Chest , age≥65	Current triage Guideline1[1]	Current triage Guideline 2[2]	Current triage Guidelines 3[3]	Alternative triage guideline	Light GBM	Lasso	Current triage guideline1	Current triage guideline2	Current triage guideline3	Alternative triage guideline	Light GBM	Lasso
Sensitivity	0.25	0.25	0.25	0.5	1	0.5	0	0	0	0.5	0.5	0.5
Specificity	0.938	0.903	0.876	0.655	0.807	0.807	0.985	0.941	0.941	0.794	0.882	0.882
Precision	0.1	0.067	0.053	0.038	0.125	0.067	0	0	0	0.067	0.111	0.111
F1 Score	0.143	0.105	0.087	0.071	0.222	0.118	0	0	0	0.118	0.182	0.182
F2 score	0.192	0.161	0.143	0.147	0.417	0.217	0	0	0	0.217	0.294	0.294
Accuracy	0.919	0.886	0.859	0.651	0.812	0.799	0.957	0.914	0.914	0.786	0.871	0.871

1) using First Fire Department Assessment variables. 2) using In-hospital Assessment 3) criteria meeting either 1) or 2) exceeds the threshold

Supplementary table S6. Comparative Accuracy of Alternative Triage Guidelines Versus Current Guidelines in Predicting ICU Admission for patients aged under 65-year-old Chest injury

Target	ICU admission											
	Training set (n= 994)						Test set (n= 422)					
Chest , age<65	Current triage Guideline1[1]	Current triage Guideline 2[2]	Current triage Guidelines 3[3]	Alternative triage guideline	Light GBM	Lasso	Current triage guideline1	Current triage guideline2	Current triage guideline3	Alternative triage guideline	Light GBM	Lasso
Sensitivity	0.188	0.219	0.302	0.562	0.885	0.781	0.087	0.087	0.13	0.696	0.783	0.696
Specificity	0.979	0.972	0.959	0.839	0.676	0.643	0.987	0.965	0.957	0.835	0.699	0.682
Precision	0.486	0.457	0.439	0.271	0.226	0.189	0.286	0.125	0.15	0.195	0.13	0.112
F1 Score	0.271	0.296	0.358	0.366	0.36	0.305	0.133	0.103	0.14	0.305	0.224	0.193
F2 score	0.214	0.244	0.322	0.463	0.559	0.481	0.101	0.093	0.134	0.46	0.391	0.34
Accuracy	0.902	0.899	0.895	0.812	0.696	0.656	0.938	0.917	0.912	0.827	0.704	0.682
	Post-hospitalization mortality											
	Training set (n= 266)						Test set (n= 82)					
Chest , age<65	Current triage Guideline1[1]	Current triage Guideline 2[2]	Current triage Guidelines 3[3]	Alternative triage guideline	Light GBM	Lasso	Current triage guideline1	Current triage guideline2	Current triage guideline3	Alternative triage guideline	Light GBM	Lasso
Sensitivity	0.4	0.6	0.6	1	1	1	0	1	1	1	1	1
Specificity	0.92	0.897	0.854	0.682	0.709	0.678	0.938	0.938	0.914	0.58	0.753	0.741
Precision	0.087	0.1	0.073	0.057	0.062	0.056	0	0.167	0.125	0.029	0.048	0.045
F1 Score	0.143	0.171	0.13	0.108	0.116	0.106	0	0.286	0.222	0.056	0.091	0.087
F2 score	0.233	0.3	0.246	0.231	0.248	0.229	0	0.5	0.417	0.128	0.2	0.192
Accuracy	0.91	0.891	0.85	0.688	0.714	0.684	0.927	0.939	0.915	0.585	0.756	0.744

1) using First Fire Department Assessment variables. 2) using In-hospital Assessment 3) criteria meeting either 1) or 2) exceeds the threshold

Supplementary table S7. Comparative Accuracy of Alternative Triage Guidelines Versus Current Guidelines in Predicting ICU Admission for Patients Aged 65 and Older with Abdominal Pelvic injury

Target	ICU admission											
	Training set (n=170)						Test set (n=57)					
Abdominal Pelvic , age≥65	Current triage Guideline1[1]	Current triage Guideline 2[2]	Current triage Guidelines 3[3]	Alternative triage guideline	Light GBM	Lasso	Current triage guideline1	Current triage guideline2	Current triage guideline3	Alternative triage guideline	Light GBM	Lasso
Sensitivity	0.31	0.448	0.552	0.621	0.724	0.586	0.125	0.125	0.125	0.5	0.5	0.375
Specificity	1	0.979	0.979	0.851	0.851	0.794	1	0.98	0.98	0.898	0.837	0.857
Precision	1	0.812	0.842	0.462	0.5	0.37	1	0.5	0.5	0.444	0.333	0.3
F1 Score	0.474	0.578	0.667	0.529	0.592	0.453	0.222	0.2	0.2	0.471	0.4	0.333
F2 score	0.36	0.492	0.593	0.581	0.665	0.525	0.152	0.147	0.147	0.488	0.455	0.357
Accuracy	0.882	0.888	0.906	0.812	0.829	0.759	0.877	0.86	0.86	0.842	0.789	0.789
	Post-hospitalization mortality											
	Training set (n= 66)						Test set (n= 26)					
Abdominal Pelvic , age≥65	Current triage Guideline1[1]	Current triage Guideline 2[2]	Current triage Guidelines 3[3]	Alternative triage guideline	Light GBM	Lasso	Current triage guideline1	Current triage guideline2	Current triage guideline3	Alternative triage guideline	Light GBM	Lasso
Sensitivity	0.333	0.667	0.667	0.667	0.667	0.667	0	0	0	0	0	0
Specificity	0.889	0.841	0.794	0.635	0.794	0.746	0.96	0.92	0.92	0.76	0.84	0.88
Precision	0.125	0.167	0.133	0.08	0.133	0.111	0	0	0	0	0	0
F1 Score	0.182	0.267	0.222	0.143	0.222	0.19	0	0	0	0	0	0
F2 score	0.25	0.417	0.37	0.27	0.37	0.333	0	0	0	0	0	0
Accuracy	0.864	0.833	0.788	0.636	0.788	0.742	0.923	0.885	0.885	0.731	0.808	0.846

1) using First Fire Department Assessment variables. 2) using In-hospital Assessment 3) criteria meeting either 1) or 2) exceeds the threshold

Supplementary table S8. Comparative Accuracy of Alternative Triage Guidelines Versus Current Guidelines in Predicting Post-hospitalization mortality for Patients Aged under 65 years old with Abdominal Pelvic injury

Target	ICU admission											
	Training set (n=484)						Test set (n=187)					
Abdominal Pelvic , age<65	Current triage Guideline1[1]	Current triage Guideline 2[2]	Current triage Guidelines 3[3]	Alternative triage guideline	Light GBM	Lasso	Current triage guideline1	Current triage guideline2	Current triage guideline3	Alternative triage guideline	Light GBM	Lasso
Sensitivity	0.222	0.284	0.358	0.63	0.716	0.481	0.28	0.2	0.4	0.6	0.64	0.4
Specificity	0.968	0.943	0.928	0.777	0.769	0.754	0.969	0.963	0.944	0.765	0.765	0.71
Precision	0.581	0.5	0.5	0.362	0.384	0.283	0.583	0.455	0.526	0.283	0.296	0.175
F1 Score	0.321	0.362	0.417	0.459	0.5	0.356	0.378	0.278	0.455	0.385	0.405	0.244
F2 score	0.254	0.311	0.38	0.548	0.611	0.422	0.312	0.225	0.42	0.49	0.519	0.318
Accuracy	0.843	0.833	0.833	0.752	0.76	0.709	0.877	0.861	0.872	0.743	0.749	0.668
	Post-hospitalization mortality											
	Training set (n=156)						Test set (n=55)					
Abdominal Pelvic , age<65	Current triage Guideline1[1]	Current triage Guideline 2[2]	Current triage Guidelines 3[3]	Alternative triage guideline	Light GBM	Lasso	Current triage guideline1	Current triage guideline2	Current triage guideline3	Alternative triage guideline	Light GBM	Lasso
Sensitivity	0.6	0.8	0.8	1	1	1	1	1	1	1	1	1
Specificity	0.861	0.821	0.768	0.55	0.755	0.781	0.815	0.889	0.759	0.556	0.778	0.778
Precision	0.125	0.129	0.103	0.068	0.119	0.132	0.091	0.143	0.071	0.04	0.077	0.077
F1 Score	0.207	0.222	0.182	0.128	0.213	0.233	0.167	0.25	0.133	0.077	0.143	0.143
F2 score	0.341	0.392	0.339	0.269	0.403	0.431	0.333	0.455	0.278	0.172	0.294	0.294
Accuracy	0.853	0.821	0.769	0.564	0.763	0.788	0.818	0.891	0.764	0.564	0.782	0.782

1) using First Fire Department Assessment variables. 2) using In-hospital Assessment 3) criteria meeting either 1) or 2) exceeds the threshold

Supplementary table S9. Comparative Accuracy of Alternative Triage Guidelines Versus Current Guidelines in Predicting ICU Admission for Patients Aged 65 and older with extremity

Target	ICU admission											
	Training set (n= 6,385)						Test set (n= 2,816)					
extremity , age≥65	Current triage Guideline1[1]	Current triage Guideline 2[2]	Current triage Guidelines 3[3]	Alternative triage guideline	Light GBM	Lasso	Current triage guideline1	Current triage guideline2	Current triage guideline3	Alternative triage guideline	Light GBM	Lasso
Sensitivity	0.168	0.261	0.304	0.467	0.576	0.484	0.224	0.255	0.327	0.418	0.51	0.469
Specificity	0.991	0.974	0.967	0.878	0.89	0.872	0.99	0.974	0.968	0.872	0.889	0.876
Precision	0.348	0.231	0.217	0.102	0.135	0.101	0.44	0.258	0.267	0.105	0.142	0.12
F1 Score	0.227	0.245	0.253	0.167	0.218	0.167	0.297	0.256	0.294	0.168	0.222	0.192
F2 score	0.188	0.254	0.282	0.272	0.348	0.275	0.249	0.256	0.312	0.262	0.336	0.297
Accuracy	0.967	0.954	0.948	0.866	0.881	0.86	0.963	0.949	0.945	0.856	0.875	0.862
	Post-hospitalization mortality											
	Training set (n= 2,276)						Test set (n= 1,012)					
extremity , age≥65	Current triage Guideline1[1]	Current triage Guideline 2[2]	Current triage Guidelines 3[3]	Alternative triage guideline	Light GBM	Lasso	Current triage guideline1	Current triage guideline2	Current triage guideline3	Alternative triage guideline	Light GBM	Lasso
Sensitivity	0.092	0.197	0.211	0.382	0.408	0.395	0.176	0.265	0.324	0.471	0.471	0.412
Specificity	0.983	0.96	0.951	0.843	0.931	0.929	0.976	0.962	0.95	0.834	0.931	0.908
Precision	0.156	0.146	0.129	0.078	0.17	0.16	0.207	0.196	0.183	0.09	0.193	0.135
F1 Score	0.116	0.168	0.16	0.129	0.24	0.228	0.19	0.225	0.234	0.151	0.274	0.203
F2 score	0.1	0.184	0.187	0.214	0.319	0.305	0.182	0.247	0.281	0.255	0.365	0.292
Accuracy	0.953	0.935	0.926	0.828	0.914	0.911	0.95	0.939	0.929	0.822	0.916	0.891

1) using First Fire Department Assessment variables. 2) using In-hospital Assessment 3) criteria meeting either 1) or 2) exceeds the threshold

Supplementary table S10. Comparative Accuracy of Alternative Triage Guidelines Versus Current Guidelines in Predicting Post-hospitalization mortality for Patients Aged under 65 years old with extremity

Target	ICU admission											
	Training set (n=9,359)						Test set (n=4,016)					
Extremity, age<65	Current triage Guideline1[1]	Current triage Guideline 2[2]	Current triage Guidelines 3[3]	Alternative triage guideline	Light GBM	Lasso	Current triage guideline1	Current triage guideline2	Current triage guideline3	Alternative triage guideline	Light GBM	Lasso
Sensitivity	0.278	0.343	0.391	0.56	0.77	0.669	0.278	0.296	0.38	0.63	0.704	0.583
Specificity	0.983	0.978	0.967	0.843	0.778	0.752	0.982	0.974	0.964	0.844	0.775	0.751
Precision	0.305	0.294	0.244	0.089	0.086	0.068	0.294	0.242	0.228	0.101	0.08	0.061
F1 Score	0.291	0.317	0.3	0.153	0.155	0.124	0.286	0.267	0.285	0.173	0.143	0.11
F2 score	0.283	0.332	0.349	0.271	0.298	0.243	0.281	0.284	0.335	0.307	0.274	0.215
Accuracy	0.964	0.961	0.952	0.836	0.778	0.749	0.963	0.956	0.949	0.839	0.773	0.747
	Post-hospitalization mortality											
	Training set (n=1,497)						Test set (n=662)					
Extremity, age<65	Current triage Guideline1[1]	Current triage Guideline 2[2]	Current triage Guidelines 3[3]	Alternative triage guideline	Light GBM	Lasso	Current triage guideline1	Current triage guideline2	Current triage guideline3	Alternative triage guideline	Light GBM	Lasso
Sensitivity	0.417	0.667	0.667	0.833	0.75	0.75	1	1	1	1	1	1
Specificity	0.939	0.929	0.906	0.77	0.851	0.801	0.936	0.939	0.911	0.733	0.863	0.837
Precision	0.052	0.07	0.054	0.028	0.039	0.03	0.143	0.149	0.108	0.038	0.072	0.061
F1 Score	0.093	0.127	0.101	0.055	0.074	0.057	0.25	0.259	0.194	0.074	0.135	0.116
F2 score	0.174	0.247	0.205	0.125	0.161	0.128	0.455	0.467	0.376	0.167	0.28	0.246
Accuracy	0.935	0.927	0.904	0.771	0.85	0.801	0.937	0.94	0.912	0.736	0.864	0.838

1) using First Fire Department Assessment variables. 2) using In-hospital Assessment 3) criteria meeting either 1) or 2) exceeds the threshold
